# Novel method for the genomic analysis of *PKD1* mutation in autosomal dominant polycystic kidney disease

**DOI:** 10.3389/fcell.2022.937580

**Published:** 2023-01-09

**Authors:** Shunlai Shang, Chao Wang, Lang Chen, Wanjun Shen, Yuansheng Xie, Wenge Li, Qinggang Li

**Affiliations:** ^1^ Department of Nephrology, China-Japan Friendship Hospital, Beijing, China; ^2^ Department of Nephrology, Chinese PLA General Hospital, Medical School of Chinese PLA, Chinese PLA Institute of Nephrology, State Key Laboratory of Kidney Diseases, National Clinical Research Center for Kidney Diseases, Beijing, China; ^3^ School of Medicine, Nankai University, Tianjin, China; ^4^ Clinical Medical School, Guangdong Pharmaceutical University, Guangzhou, China

**Keywords:** ADPKD, PKD1, mPCR, MLPA (multiplex ligation-dependent probe amplification), targeted region sequencing

## Abstract

Autosomal dominant polycystic kidney disease (ADPKD) is the most common inherited kidney disease. Although next-generation sequencing (NGS) technology can be used to sequence tens of thousands of DNA molecules simultaneously. It has poor capture efficiency for the six *PKD1* pseudogenes and GC-rich regions. Multiplex ligation-dependent probe amplification (MLPA) technology can detect consecutive deletions of exons, but it is less sensitive for single-base mutations. However, pathogenic genes might not be detected in some patients, even when using the above methods. Improving the detection rate of pathogenic genes is an important technical problem hindering clinical diagnosis of ADPKD. Four pedigrees of ADPKD patients with mutation sites not identified by NGS were examined by other methods. First, MLPA was performed. Then, pedigrees in which MLPA did not identify pathogenic genes were subjected to multiplex polymerase chain reaction (MPCR) and targeted region sequencing. Finally, the detected mutation sites were verified by Sanger sequencing. The results showed that MLPA detected the following *PKD1* exonic deletion mutations in three pedigrees: PKD1-18 nt–290 nt, PKD1-up-257 nt, PKD1-up-444 nt and PKD1-3 nt–141 nt. A new mutation site was identified through targeted region sequencing in one pedigree: PKD1 NM_001009944: c.151T > C at the protein level, described as p. Cys51Arg. In summary, we established a system of genetic detection and analytical methods, from NGS to MLPA to targeted region sequencing and finally to Sanger sequencing. We combined MPCR and targeted region sequencing for the first time in ADPKD diagnosis, which further improved diagnosis accuracy. Moreover, we identified one new missense mutation and four new deletion mutations.

## Introduction

Autosomal dominant polycystic kidney disease (ADPKD) is the most common hereditary kidney disease, with a prevalence of approximately 1/400–1/1,000; ADPKD manifests as multiple progressive cysts in both kidneys, which will eventually destroy the structure and function, leading to end-stage renal disease (ESRD) and a variety of extrarenal manifestations, such as hypertension, liver and pancreatic cysts, intracranial aneurysms, abdominal hernias, and heart valvular disease (Lanktree et al., 2021). *PKD1* and *PKD2* gene mutations are the most common cause of ADPKD. The pathogenic gene is *PKD1* in approximately 85% of patients, and *PKD2* in approximately 15% (Cornec-Le Gall et al., 2018; Hardy and Tsiokas, 2020; Lu et al., 2020). Some studies have shown that compared with *PKD2*-mutated patients, in *PKD1*-mutated patients, the glomerular filtration rate decreases faster, ESRD occurs earlier, and morbidity and mortality are higher (Hateboer et al., 1999; Kurashige et al., 2015). Because ADPKD is a serious disease that often involves multiple tissues and organs and there is no effective treatment method at present, early diagnosis and screening of ADPKD are particularly important. Ultrasound examination is the most commonly used clinical diagnostic method for ADPKD. Detecting lesions such as enlarged kidneys and cysts, but its sensitivity and specificity are not high, especially for the population without symptom onset. Genetic testing can clearly identify mutant genes through linkage analysis of family members, can diagnose patients early, can provide evidence for prenatal testing. Genetic testing is also helpful for implementing clinical intervention as soon as possible, correcting risk factors, detecting and treating complications early, and improving prognosis.

Commonly used genetic detection techniques for polycystic kidney disease (PKD) include Sanger sequencing, next-generation sequencing (NGS) (Kinoshita et al., 2016), and multiplex ligation-dependent probe amplification (MLPA). Traditional Sanger sequencing is commonly employed to detect PKD, but the throughput of this method is low, and the workload is heavy. Because *PKD1* contains 46 exons and multiple complex repetitive regions, Sanger sequencing is not sufficient for mutation analysis of this gene. NGS, which is a high-throughput sequencing technique, mainly includes whole-exome sequencing (WES) (LaDuca et al., 2017), target capture sequencing (Panel), and whole-genome sequencing. These methods can be used to simultaneously sequence tens of thousands of DNA molecules, with advantages in speed, accuracy, sensitivity, and coverage. In MLPA, probes hybridize to target sequence DNAs. After probe-specific ligation, the hybridized products are amplified by polymerase chain reaction (PCR), and the PCR product is separated by capillary electrophoresis, the data are collected, and analyzed using specific analysis software (Lu et al., 2020). MLPA can be applied to detect consecutive deletions or duplicate mutations of exons but has a low sensitivity for single-base mutations. Multiplex PCR (MPCR) is a novel PCR amplification technique that is an improvement over conventional PCR. Two or more pairs of primers can be added to one reaction system to simultaneously amplify multiple nucleic acid fragments to increase the detection rate and identify mutations and their types. MPCR has the advantages of high efficiency, systematicity, economy, and simplicity.

In general, application of MPCR for ADPKD diagnosis can simplify analysis of the *PKD1* and *PKD2* genes, which is extremely important for early screening of ADPKD pedigrees and prenatal diagnosis (Deng et al., 2022). This study comprehensively detected and analyzed patients with PKD and their relatives through a combination of several different sequencing methods and MPCR, yielding possible genetic etiologies of ADPKD.

## 2 Methods

### 2 1 Sample collection and DNA extraction

The participating patients and their immediate family members signed the informed consent, 2 ml of peripheral venous blood was collected from each subject and placed in an ethylenediaminetetraacetic acid anticoagulation tube. Genomic DNA was extracted from whole blood using the QIAamp DNA Mini Kit (Qiagen, Shanghai, China, 180134) following the manufacturer’s instructions. The DNA was quantified in a Nanodrop 2000 (Thermo Fisher Scientific, DE) (Diefenbach et al., 2018; Shang et al., 2019).

### 2.2 NGS technique

A 3 µg sample of DNA from each subject was fragmented using a Covaris S2 ultrasonic instrument (Covaris, United States). The ends of the DNA segments were repaired and linked with adapters. The length of the mature library produced was approximately 320 bp–400 bp. The library was amplified, and DNA samples were quality-controlled using the Nanodrop 2000 sample quantitative detector (Thermo Fisher Technology Co., Ltd., United States) and Agilent 2,100 Bioanalyzer (Agilent Technology Company, United States). A DNA library for the Illumina second-generation sequencing platform was efficiently prepared using the GenCap^®^ second-generation sequencing rapid DNA library construction kit (Illumina). GenCap^®^ liquid phase target gene capture technology (Beijing MyGenostics Co. Ltd., China) was employed to capture the relevant panel or whole-exome regions (Xu et al., 2020; Li A et al., 2015). Dual-end sequencing of the captured regions with a read length of 150 bp was performed with an Illumina NextSeq 500 second-generation sequencer (Baert-Desurmont et al., 2018).

### 2.3 Data screening and bioinformatic analysis

After sequencing, low-quality variations were filtered out using a quality score ≥ 20, and Burrows‒Wheeler aligner software (Li and Durbin, 2009) was used to align the clean reads to the reference human genome (hg19). Single nucleotide polymorphisms (SNPs) and insertions or deletions (InDels) were identified using Genome Analysis Toolkit software, and those with a frequency ≥ 0.05 in the 1,000 Genomes Project, ESP6500, and ExAC databases were removed. Non-synonymous variants were evaluated using four algorithms, namely, SIFT, PolyPhen-2, Mutation-Taster, and GERP++, to predict pathogenicity (Table 1).

**TABLE 1:** Software and database.

Number	Name	Website
1	BWA	http://bio-bwa.sourceforge.net/
2	GATK	https://software.broadinstitute.org/gatk/
3	1000 genome	http://www.1000genomes.or/
4	EVS	http://evs.gs.washington.edu/EVS
5	dbSNP	http://www.ncbi.nlm.nih.gov/projects/SNP/
6	EXAC	http://exac.broadinstitute.org/
7	HGMD	http://www.biobase-international.com/product/hgmd/
8	SIFT	http://sift.jcvi.org/
9	PolyPhen-2	http://genetics.bwh.harvard.edu/pph2/
10	MutationTaster	http://www.mutationtaster.org/

### 2.4 MLPA

Fragment deletion was identified by MLPA. The SALSA MLPA kit is commercially available from MRC-Holland (Amsterdam, the Netherlands; catalog number MLPA p351-025R salsa mlpa probemix p351 pkd1-25rxn and p352-025R salsa mlpa probemix p352 pkd1-pkd2-25rxn). Deletion/duplication analysis of the *PKD1* exons was performed according to the manufacturer’s instructions. The results of MLPA testing of the *PKD1* gene showed deletion of exons. It is generally considered normal that the fluorescence signal intensity is between 0.75 and 1.3.

### 2.5 Long-range PCR

We designed LR-PCR primers to amplify DNA fragments by PCR. The LR-PCR products were purified using the Agencourt AMPure XP kit (Beckman Coulter, Inc., Brea, CA, United States), followed by quantification and fragmentation using a NEBNext Fast DNA Fragmentation Kit (New England BioLabs, Ipswich, MA). Sequencing was performed with an ABI3730xl sequencer (Applied Biosystems, United States).

### 2.6 MPCR + targeted region sequencing

The gene primers were designed using Primer 5.0. The first primer sequence of the targeted region (C.151T upstream and downstream 100 bp) was CGGGCCCCGCCTGAGCT TGT​GGC​GTC​CGC​GGG​GAT, and the second primer sequence was TAT​TTA​GCA​GGG​CCG​CCG​TAT​GCC​AGT​CCC​TCA​TCG​C. Using the multiple PCR amplification kit KT109 (Tiangen, China), genomic DNA was amplified by MPCR. The amplified product was purified. T4 ligase, T4 alkaline phosphatase, and Klenow fragment were applied to the purified DNA. The details are as follows: 1) The primary PCR amplification mixture system (Supplementary Table S1) was prepared; 2) After shaking and centrifuging, the samples were placed into the PCR instrument, and the procedure was carried out (Supplementary Table S2); 3) After PCR, agarose gel electrophoresis was performed (2% agarose gel, 100 bpm, 140 V, run for 20 min), and at least one specific band could be seen in the PCR products detected at approximately 200 bp; 4) PCR product purification was carried out according to the ratio of magnetic beads:sample volume ratio = 1:1, the samples were washed twice with 180 µl 80% EtOH, eluted with 30 µl enzyme-free water, and 28 µl was removed for later use; 5) The secondary PCR amplification mixture system (Supplementary Table S3) was prepared; 6) After centrifugation, the samples were placed into the PCR instrument, and the procedure was run (Supplementary Table S4); 7) PCR products were purified according to the ratio of magnetic beads: sample volume = 1:1, washed twice with 180 µl 80% EtOH, eluted with 30 µl enzyme-free water, and 28 µl was removed for later use; and 8) The remaining 2 µl sample was used for agarose gel electrophoresis (100 bp marker, 1% agarose gel, 140 V, run for 15 min). Fragments were end-repaired, and A bases were added to the 3′ ends of the fragments. Fragments were then ligated to form a standard Solexa sequencing library. The libraries were sequenced on an Illumina NextSeq 500. The first amplicon (287 bp) and second amplicon (699 bp) of the MPCR/target region are shown in Supplementary Tables S5, S6, respectively. The *PKD1* gene is illustrated in Supplementary Figure S1, showing the regions targeted by the MPCR and LR-PCR strategies.

### 2.7 Sanger sequencing verification

The primers for Sanger sequencing were synthesized according to the DNA fragments to be sequenced. The DNA was amplified by PCR and sequenced by Sanger sequencing on an ABI3730xl sequencer (Applied Biosystems, United States). The sequencing results were aligned and analyzed with the reference sequence using “Mutation Surveyor” software (Richards et al., 2015).

## 3 Results

**FIGURE 1:**
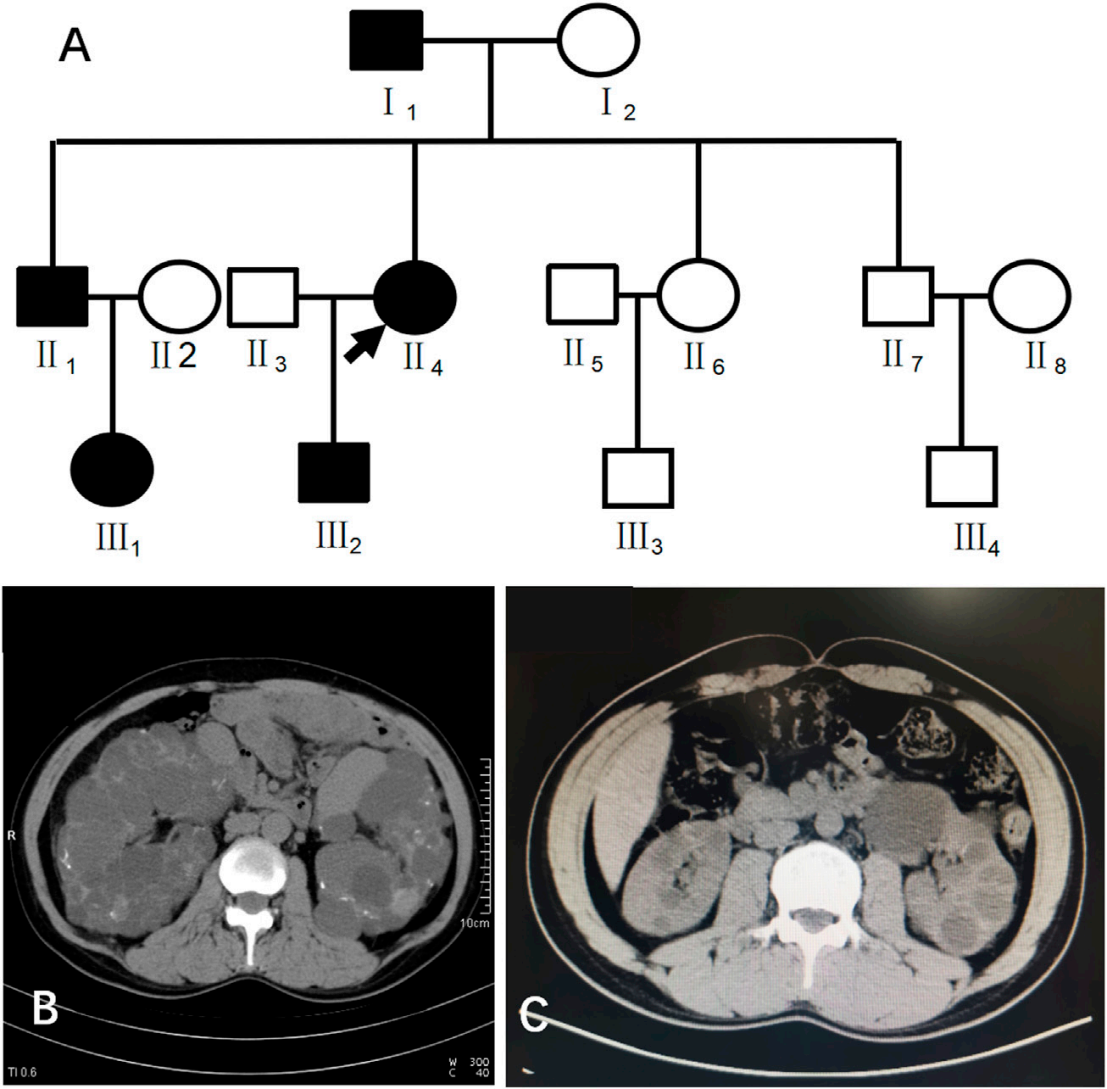
**(A)** shows the pedigree of Case 1 (III_2_). Arrow indicates the proband. **(B)** shows the CT image of II_4_. C shows the CT image of III_2_.

Case 1 (III_2_ in Figure 1) was a 28-year-old male with creatinine level 92 μmol/L and blood pressure of 130/80 mmHg. A CT scan of his kidneys in 2019 showed that both kidneys were not enlarged in size but had irregular morphology, and the parenchyma of both kidneys showed multiple water-like density shadows of different sizes smaller than 47 mm × 49 mm and with clear boundaries. Some lesions showed slightly high-density strips on the edges, and the bilateral renal sinus areas showed small dot-like dense shadows, suggesting bilateral polycystic kidneys and some cystic lesions that were suspected to be complex cysts (Figure 1).

This patient had a family history of PKD. His family pedigree showed the following (Figure 1) I_1_, 78 years old, had a polycystic kidney and polycystic liver with a history of hypertension and hemodialysis for 1 year; II_1_, 52 years old, had bilateral polycystic kidneys with a history of hypertension and creatinine 75 μmol/L; II_4_, 51 years old, had bilateral polycystic kidneys with suspected partial intracystic hemorrhage, multiple hepatic cysts, multiple uterine cysts (Figure 1), a history of hypertension, and hemodialysis starting at the age of 45; and III_1_, 26 years old, had one polycystic kidney, creatinine 38.16 μmol/L, and negative urine protein and occult blood.

NGS detection in case 1 did not identify clear pathogenic point mutations related to the disease. In NGS analysis, we found that the fraction of target genes covered by at least 20 × was 95% or more, and the average sequencing depth on the target was 100 × or more. To evaluate the presence ofmicrodeletion or duplication mutations in relevant gene fragments, we then performed MLPA detection but still did not detect clear copy number variations in the exon segments of the *PKD1* or *PKD2* gene. Next, we reanalyzed the raw data for *PKD1*, *PKD2*, and related genes and found that due to the low sequencing depth, the coverage of NM_001009944: c.151T > C, where the *PKD1* gene is located, was unsatisfactory. However, the results were still suspicious, and *PKD1* gene mutations could not be completely ruled out.

**FIGURE 2:**
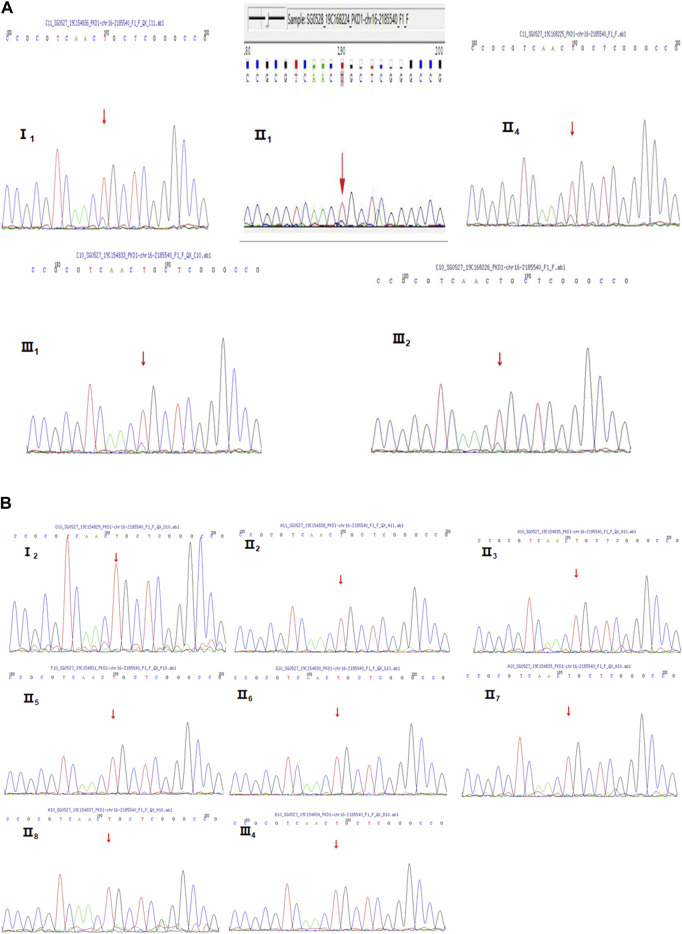
Sanger sequencing diagrams of the Case 1 pedigree. **(A)**, III_2_, II_4_, I_1_, II_1_, and III_1_ show the heterozygous mutation at NM_001009944:c.151T>C. The red arrow indicates the mutation position. **(B)**, II_3_, I_2_, II_6_, II_5_, III_3_, III_4_, II_7_, II_8_, and II_2_ indicate that there is no mutation in NM_001009944:c.151T>C. There is no mutation at the position indicated by the red arrow.

Because of multiple amplification failures due to the high GC content of the amplicons, we abandoned the LR-PCR-Sanger sequencing strategy and applied. MPCR and NGS sequencing, performed as follows. We optimized the process by redesigning the primers and changing the amplification parameters and performed deep (10,000 ×) sequencing of the target region after MPCR amplification and eventually verified the heterozygous mutation NM_001009944:c.151T > C. We then verified this locus in II_1_ and II_4_ using this method, and the results are shown in Table 2. Additionally, the allele frequency and pathogenicity predictions of this locus are presented in Supplementary Table S7. We found that all three patients harbored the same mutation. Finally, Sanger sequencing was performed for the other five members of the family, and all family members with similar symptoms were found to carry the mutation, whereas healthy family members did not (Figure 2), the results are consistent with pedigree cosegregation.

**TABLE 2:** MPCR + targeted region sequencing results of the case 1 pedigree.

Patient	Gene	Mutation site	Transcript; exon	Nucleotide changes (amino acids)	Normal/mutation (mutation ratio)
Ⅲ_2_	PKD1	Chr16:2185540	NM_001009944; exon1	c.151T>C (p.C51R)	128/118 (48%)
Ⅱ_4_	PKD1	Chr16:2185540	NM_001009944; exon1	c.151T>C (p.C51R)	31/29 (48%)
Ⅱ_1_	PKD1	Chr16:2185540	NM_001009944; exon1	c.151T>C (p.C51R)	29/46 (61%)

**FIGURE 3:**
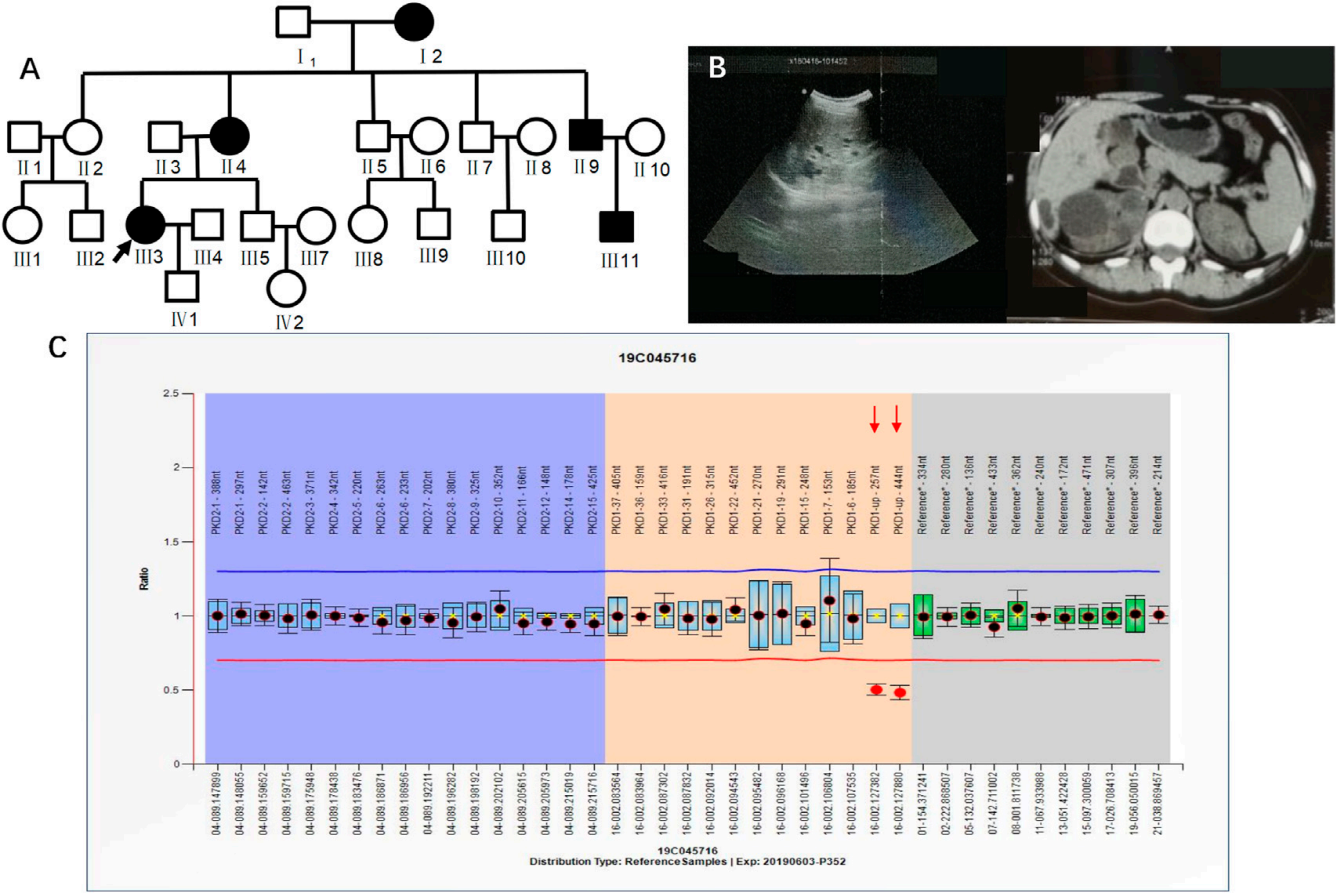
**A)** shows the pedigree of Case 2 (III_3_), and the arrow indicates the proband. **(B)** Imaging examination results (ultrasound and MRI) of III_3_. **(C)** MLPA result of III_3_ (fluorescence signal intensity between 0.7 and 1.3 is considered normal).

Case 2 (III_3_ of Figure 3) was a 36-year-old female with blood pressure 144/95 mmHg and creatinine 70 μmol/L. CT in 2015 showed polycystic kidneys and polycystic liver. The patient was followed regularly every year. Ultrasound in 2018 showed that both kidneys were enlarged, with a plump morphology and multiple cystic echoes in both kidneys. The large cyst in the right kidney was approximately 45 mm × 42 mm in size, and the large cyst in the left kidney was approximately 16 mm × 15 mm in size. CT in 2019 showed multiple round-like and unenhanced cystic density shadows of various sizes in both kidneys. The large cyst in the right kidney was approximately 49 mm × 51 mm in size, and the large cyst in the left kidney was approximately 26 mm × 26 mm. NGS sequencing showed no abnormal mutations, while MLPA detected *PKD1* upstream heterozygous deletions (PKD1-up-257 nt, PKD1-up-444 nt) (Figure 3). III_3_ had a family history of ADPKD, and I_2_, II_4_, II_9_, and III_11_ all had ADPKD (Figure 3).

**FIGURE 4:**
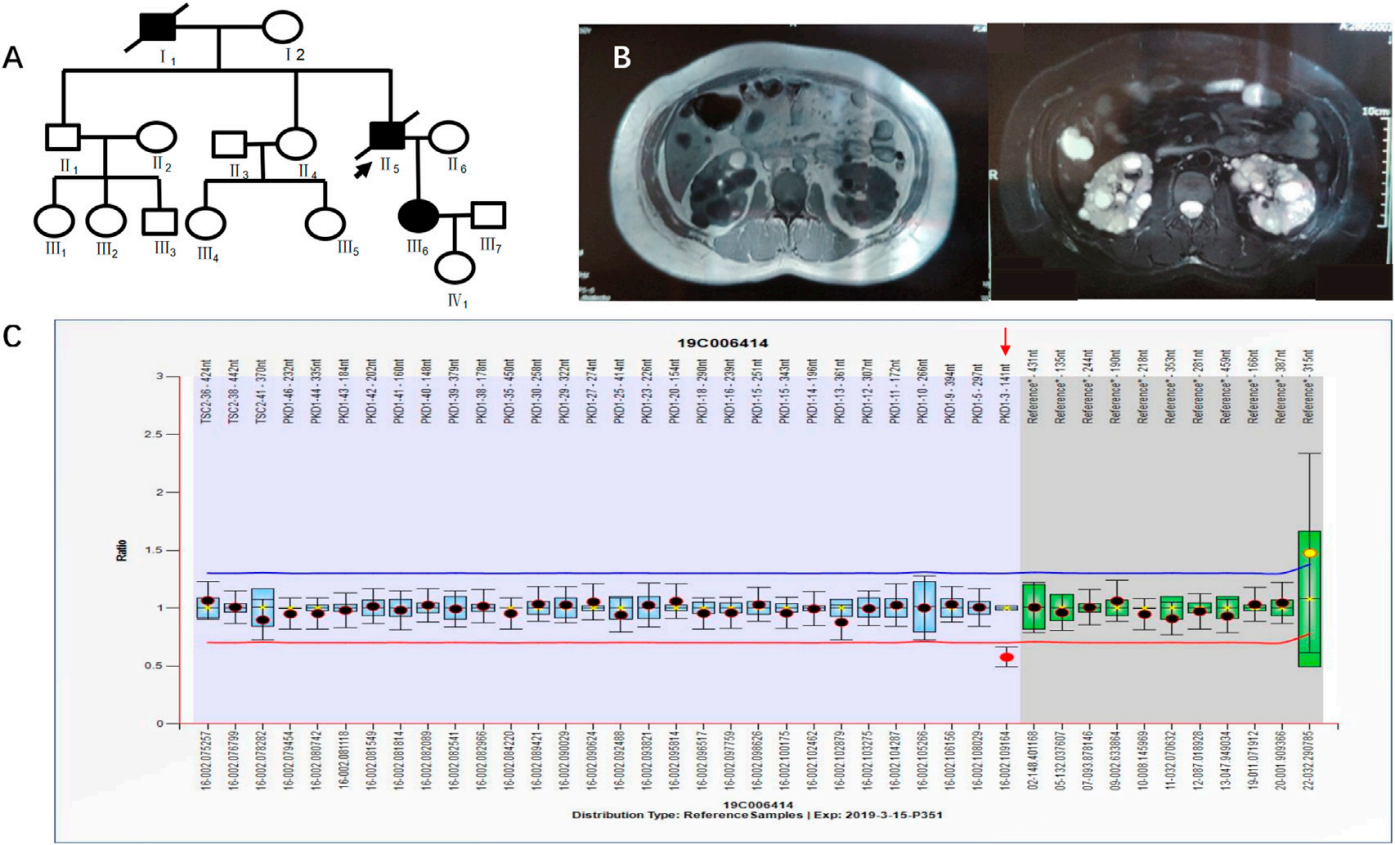
**A)** shows the pedigree of Case 3 (III_6_), and the arrow indicates the proband. **(B)** Imaging examination results (enhanced CT and MRI) of III_6_. **(C)** MLPA result of III_6_ (fluorescence signal intensity between 0.7 and 1.3 is considered normal).

Case 3 (III_6_ of Figure 4) was a 36-year-old female with blood pressure 140/96 mmHg and creatinine 79 μmol/L. Kidney magnetic resonance imaging (MRI) in 2019 showed multiple cysts in both kidneys, some complicated with bleeding, and multiple cysts in the liver. Her father was diagnosed with PKD by ultrasound and was treated by hemodialysis. He has since passed away. Her grandfather had edema and suffocation symptoms, and he died of unknown causes. NGS showed no abnormalities. MLPA results indicated a heterozygous deletion mutation in exon 3 of the *PKD1* gene (PKD1-3 nt–141 nt) (Figure 4). III_6_ had a family history of ADPKD, and I_1_ and II_5_ had ADPKD (Figure 4).

**FIGURE 5:**
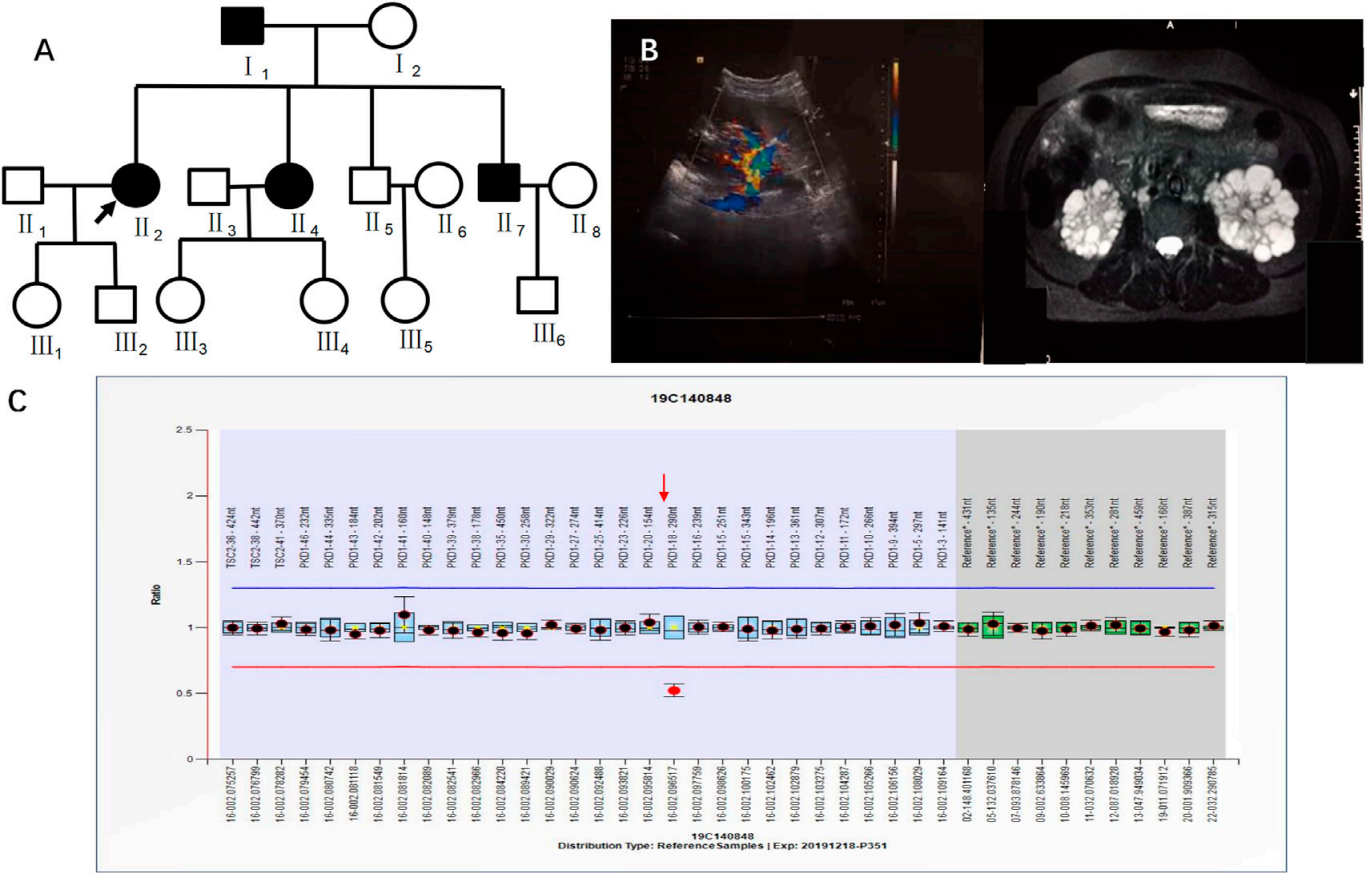
**A)** shows the pedigree of Case 4 (II_2_), and the arrow indicates the proband. **(B)** Imaging examination results (ultrasound and MRI) of II_2_. **(C)** MLPA result of II_2_ (fluorescence signal intensity between 0.7 and 1.3 is considered normal).

Case 4 (II_2_ of Figure 5) was a 41-year-old female with creatinine 55.8 μmol/L, blood pressure 150/95 mmHg, urine protein (+/-), and urinary occult blood (+). Ultrasound in 2019 showed multiple anechoic shadows in both kidneys. The large one in the right kidney was approximately 32 mm × 24 mm, and the large one in the left kidney was approximately 61 mm × 41 mm. There were multiple anechoic shadows in the liver. The large cyst was approximately 9 mm in diameter, suggesting polycystic kidneys and small hepatic cysts. Based on CT both kidneys were enlarged and had multiple round cystic density shadows. NGS did not detect clear pathogenic mutations related to the polycystic kidney phenotype, yet MLPA results indicated a heterozygous deletion mutation in exon 18 of the *PKD1* gene (PKD1-18 nt–290 nt) (Figure 5). II_2_ had a family history of ADPKD, and I_1_, II_4_ and II_7_ had ADPKD (Figure 5).

## 4 Discussion

**FIGURE 6:**
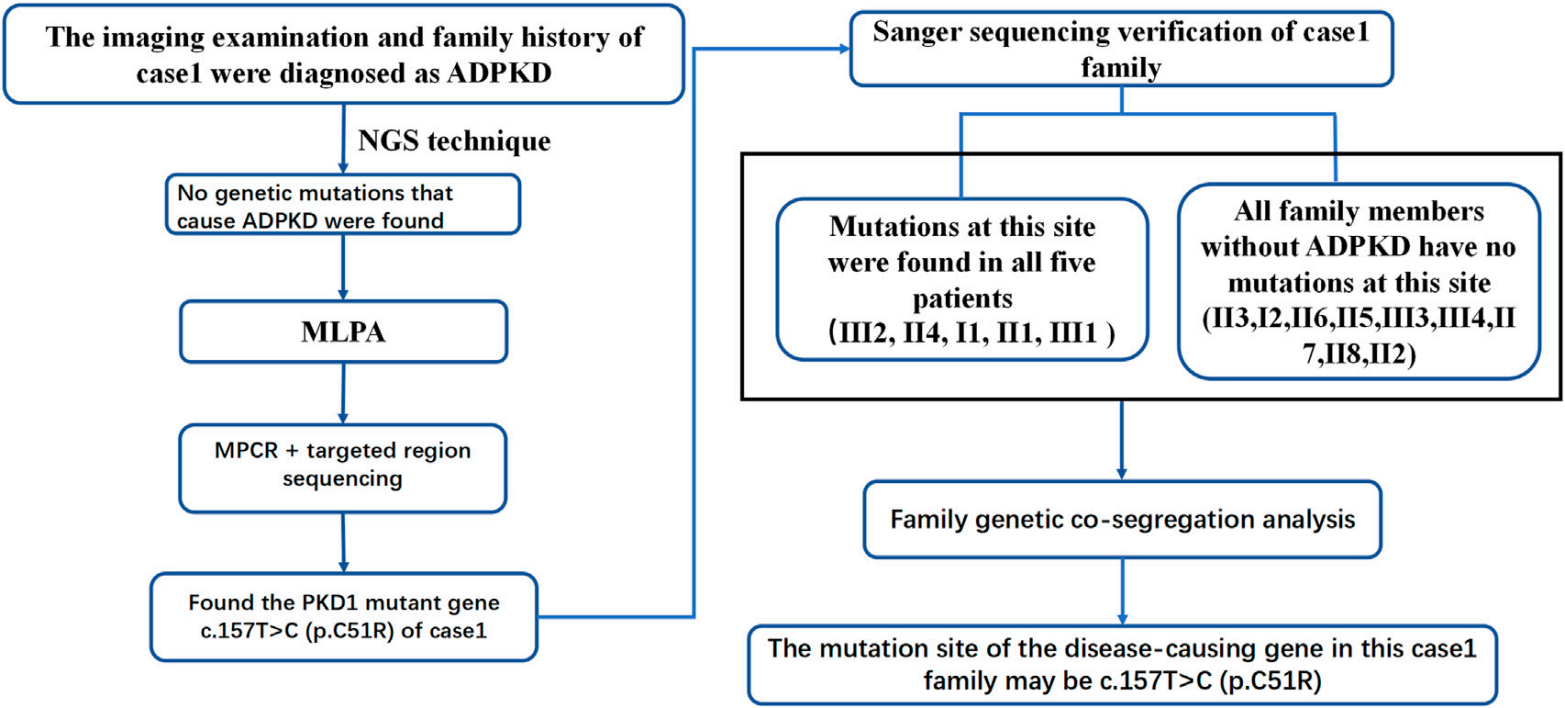
Case 1 pedigree diagnosis flowchart.

ADPKD is one of the most common genetic diseases affecting renal tubules. Approximately 85% of ADPKD patients have *PKD1* gene mutations. Genetic tests can identify the mutated genes in ADPKD patients, provide a basis for the diagnosis and treatment of ADPKD and be used to optimize prenatal and postnatal care and genetic blockade. In this study, patient 1 was diagnosed with PKD based on clinical features and imaging examinations. Although imaging features suggested possible *PKD1* gene mutations. Panel and WES sequencing did not detect any clear disease-related pathogenic point mutations. To further exclude the possibility of PKD caused by microdeletion or duplicate mutations of *PKD1* and/or other genes, MLPA was performed. As the examination still did not identify any relevant mutations, the possibility of a deletion or duplicate mutation was ruled out. Later, we performed pedigree linkage analysis on the raw WES data, which revealed four suspicious genes for screening (Supplementary Table S8 for details). However, combining those findings with database prediction analysis of the conservation, tissue specificity, and pathogenicity of these loci indicated that these four new genes were basically excluded. Instead, we analyzed the imaging characteristics of patient 1’s mother (II_4_) and still considered the possibility of *PKD1* gene mutation. Overall, diagnosing ADPKD based only on clinical manifestations and imaging characteristics is uncertain, and a supporting genetic diagnosis is needed. Accordingly we further analyzed the raw NGS data of the patient and found several suspicious sites. For example, the suspected pathogenic site NM_001009944:c.151T > C reported in the literature is considered a suspicious site (Li and Durbin, 2009). Due to poor coverage and low sequencing depth at these sites, we performed MPCR combined with 10,000 × targeted region (the target region is exons 1–10 of *PKD1*) sequencing for these suspected sites, and the results confirmed the presence of these mutations. Verification performed in II_4_ and II_1_ identified one point mutation. Sanger sequencing was performed in patient 1’s family members, and all kidney patients in this pedigree were found to have the same mutation, while no healthy member had the mutation, which is consistent with pedigree cosegregation (Figure 6).

**FIGURE 7:**
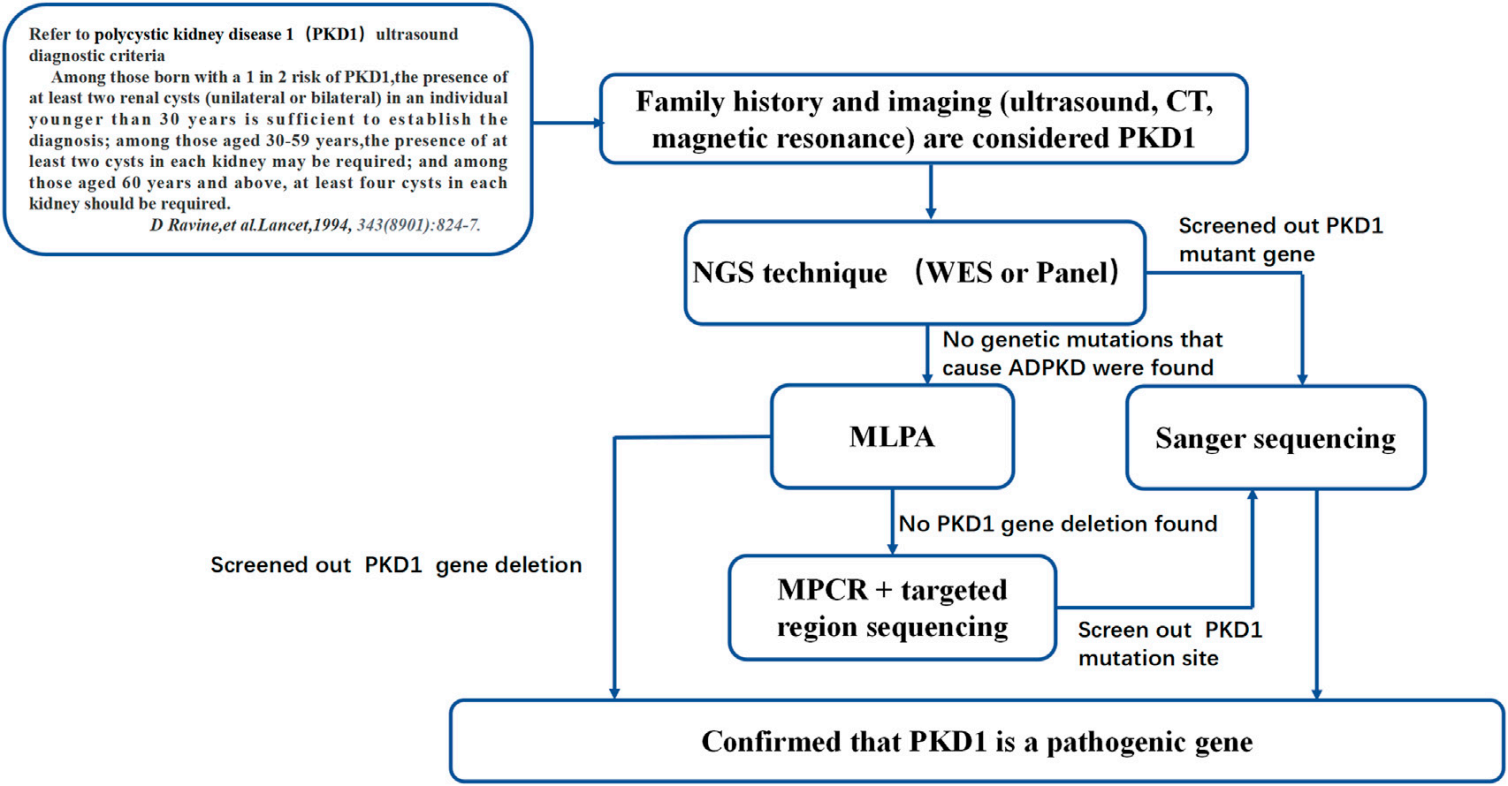
ADPKD genetic diagnosis flowchart. This diagnosis flowchart is suitable for patients who have been diagnosed with ADPKD through clinical diagnosis but have not been found to have pathogenic genes that cause ADPKD based on gene sequencing and MLPA.

The point mutation NM_001009944:c.151T > C was predicted to be harmful by the Polyphen2_HDIV, Mutation Taster, and PROVEAN prediction databases. According to the American College of Medical Genetics and Genomics (ACMG) guidelines (Richards et al., 2015), the site is likely pathogenic. The specific analysis in our study was as follows: NM_001009944:c.151T > C is not found in the Exome Sequencing Project, 1,000 Genomes or ExAC populations (PM2) (Supplementary Table S7). Through a literature review, we found one study that reported mutation of the same amino acid residue NM_001009944:p.Cys51Trp along with one nucleotide mutation at a different site c.153C > G. That study screened 700 ADPKD patients by direct sequencing, quantitative fluorescence MPCR, and array-comparative genomic hybridization (array-CGH) chip technology. The amino acid at this locus was mutated from cysteine to tryptophan. The authors suggested that this amino acid mutation is pathogenic (Audrezet et al., 2012). Our study found, through MPCR and NGS, that the amino acid at this locus was mutated from cysteine to arginine (NM_001009944:c.151T > C) in patient 1 (PM5). The mutation site meets the principle of genetic cosegregation in the Case 1 family. People with mutations at this site have PKD, and all people with no mutation at this site do not have PKD (PP1). The patient’s phenotype and family history were highly specific for a disease (ADPKD) with a single genetic etiology (PP4). This information may be added to the ADPKD gene diagnosis database. Based on the genetic diagnosis process of patient 1’s family, we summarized a sequence of genetic diagnostic procedures for ADPKD patients (Figure 7). For patients diagnosed with ADPKD based on family history and imaging diagnostic criteria who are suspected to carry gene mutations (Pei et al., 2009; Rong et al., 2010; Pei et al., 2015), NGS (including panel and WES sequencing) should be performed first. Due to the high throughput, fast sequencing speed, and wide range coverage of NGS technology, all exons of the *PKD1* and *PKD2* genes can be captured and detected. Therefore, NGS can be used to identify most *PKD1* or *PKD2* gene mutation sites (Hwang et al., 2016). Sanger sequencing verification can confirm the diagnosis. To confirm the effectiveness of the NGS-mPCR sequencing process, we used the above method to discover two *PKD1* mutation sites (c.61dupG and. c.2180T > C), Please refer to Supplementary Figure S2; Supplementary Table S9 for details.

Additionally, more cases confirmed the advantages of this method. For people without gene mutations identified by NGS sequencing, deletion or duplication of gene fragments should be considered. They can be detected by MLPA sequencing (as in Cases 2–4). MLPA is easy to perform and can be used to efficiently and accurately detect consecutive deletions or amplifications in the *PKD1* gene (Consugar et al., 2008). Failure of MLPA to identify any relevant mutations may be due to off-target effects and poor coverage of NGS sequencing of some *PKD1* gene sequences.

*PKD1* is a large gene with 46 exons located on chromosome 16 (16p13.3) (Consortium, 1994). There are six *PKD1* pseudogenes (*PKDP1-P6*) on chromosome 16 that have been six times and share high homology with *PKD1* (Bogdanova et al., 2001; Martin et al., 2004; Payne et al., 2021), and the GC content of some of these sequences is high (Al-Muhanna et al., 2019). NGS sequencing alone has a poor capture efficiency for high-GC-content regions, resulting in some *PKD1* gene mutations being undetected. To address this problem, the authors have attempted to use NGS screening after *PKD1* gene amplification with a long-range PCR technique, increasing the detection rate of *PKD1* gene mutations to approximately 90% (Rossetti et al., 2007; Rossetti et al., 2012). Nevertheless for some genes with poor data quality and microdeletion, long-range PCR is not sufficient for data analysis. This study is the first to combine MPCR amplification with targeted region sequencing (with an effective sequencing depth of 10,000×) to accurately detect suspected pathogenic sites. At present, there are many regions of *PKD1* gene with high GC, but few areas that can’t be detected by next-generation sequencing (Al-Muhanna et al., 2019). Therefore, mPCR could basically cover key areas, which is a good supplementary experiment. Due to the low economic cost of MPCR and the small number of MPCRs required, the total cost of ADPKD detection method is not too high and does not bring great financial pressure to patients (Sigmund et al., 2019).

## 5 Conclusion

This study combined NGS sequencing, MLPA sequencing, and Sanger sequencing to address the difficulties in identifying *PKD1* pseudogenes and low detection rates in high-GC-content regions. This is the first study to apply MPCR combined with targeted region sequencing for genetic detection in ADPKD. We established a novel sequence of genetic detection and analytical methods that is conducive to improving the accuracy of genetic diagnosis of ADPKD patients, and it will help to guide the diagnosis and prognosis of this diease.

## Data Availability

The data presented in the study are deposited in the https://bigd.big.ac.cn/gsa-human/browse, accession number HRA003651.
