# Supplementary material for: Novel method for the genomic analysis of PKD1 mutation in autosomal dominant polycystic kidney disease
Source: Front Cell Dev Biol. 2023 Jan 9;10:937580. doi: 10.3389/fcell.2022.937580 (PMC9868468; doi:10.3389/fcell.2022.937580)
Supplement: Supplementary file 1 [file Table1.DOCX]

Supplementary table 1 Primary PCR amplification and amplification system:

| Reagent | Volume（μL） |
| --- | --- |
| 5X Phusion HF Buffer | 10 |
| dNTP (10mM） | 1.2 |
| PKU1 Mix (10μM) | 1 |
| PKU2 Mix (10μM) | 1 |
| gDNA | 100ng |
| Phusion Hot Start II DNA Polymerase | 1 |
| DMSO | 2ul |
| ddH2O | Up to 50 μL |
