# Supplementary material for: Novel method for the genomic analysis of PKD1 mutation in autosomal dominant polycystic kidney disease
Source: Front Cell Dev Biol. 2023 Jan 9;10:937580. doi: 10.3389/fcell.2022.937580 (PMC9868468; doi:10.3389/fcell.2022.937580)
Supplement: Supplementary file 2 [file Table8.DOCX]

|  | **PLGLB1** | **PLGLB2** | **PAN3** | **OR4K1** |
| --- | --- | --- | --- | --- |
| Protein mainly expression organization | right lobe of liver, liver, cerebellum hemisphere | liver, right lobe of liver, right hemisphere of cerebellum | oviduct epithelium, secondary oocyte, pancreas. | testis and gall bladder tissues |
| Fuction | Diseases associated with PLGLB1 include Localized Chondrosarcoma and Cerebellar Ataxia Type 43 ^[1-2]^ | Diseases associated with PLGLB2 include [Plica Syndrome](https://www.malacards.org/card/plica_syndrome) and [Localized Chondrosarcoma](https://www.malacards.org/card/localized_chondrosarcoma)^[3-4]^ | Regulatory subunit of the poly(A)-nuclease (PAN) deadenylation complex, one of two cytoplasmic mRNA deadenylases involved in general and miRNA-mediated mRNA turnover^[5-7]^. | Olfactory receptors interact with odorant molecules in the nose, to initiate a neuronal response that triggers the perception of a smell^[8]^. |

Supplementary Table 8 Information about four suspicious genes

**References**

1. Ichinose A. Multiple members of the plasminogen-apolipoprotein(a) gene family associated with thrombosis. Biochemistry. 1992;31(12):3113-3118.

2. Frank SL, Klisak I, Sparkes RS, Lusis AJ. A gene homologous to plasminogen located on human chromosome 2q11-p11. Genomics. 1989;4(3):449-451.

3. Kim YC, Kim S, Kim HK, et al. Genome-Wide Association Study Identifies Eight Novel Loci for Susceptibility of Scrub Typhus and Highlights Immune-Related Signaling Pathways in Its Pathogenesis. Cells. 2021;10(3):570.

4. Ichinose A, Espling ES, Takamatsu J, et al. Two types of abnormal genes for plasminogen in families with a predisposition for thrombosis [published correction appears in Proc Natl Acad Sci USA 1991 Apr 1;88(7):2967]. Proc Natl Acad Sci U S A. 1991;88(1):115-119.

5. Uchida N, Hoshino S, Katada T. Identification of a human cytoplasmic poly(A) nuclease complex stimulated by poly(A)-binding protein. J Biol Chem. 2004;279(2):1383-1391.

6. Wolf J, Passmore LA. mRNA deadenylation by Pan2-Pan3. Biochem Soc Trans. 2014;42(1):184-187.

7. Shang J, Chen WM, Liu S, et al. CircPAN3 contributes to drug resistance in acute myeloid leukemia through regulation of autophagy. Leuk Res. 2019;85:106198.
