# Supplementary material for: Novel method for the genomic analysis of PKD1 mutation in autosomal dominant polycystic kidney disease
Source: Front Cell Dev Biol. 2023 Jan 9;10:937580. doi: 10.3389/fcell.2022.937580 (PMC9868468; doi:10.3389/fcell.2022.937580)
Supplement: Supplementary file 3 [file Table7.DOCX]

Supplementary Table 7 Allele frequency and Pathogenicity score of this variation ( c.151T>C)

| Nucleotide changes | Inhouse | esp6500siv2_all | 1000g2015aug_all | ExAC_ALL | ExAC_AFR | ExAC_AMR | ExAC_EAS | ExAC_FIN | ExAC_NFE | ExAC_OTH | ExAC_SAS | SIFT_score | MutationTaster_score | CADD_phred |
| --- | --- | --- | --- | --- | --- | --- | --- | --- | --- | --- | --- | --- | --- | --- |
| c.151T>C | - | - | - | - | - | - | - | - | - | - | - | 0.003 | 0.998 | 16.43 |

esp6500siv2_all, NHLBI Exome Sequencing Project ESP6500; SIFT_score, SIFT score (SIFTori). Scores range from 0 to 1. The smaller the score the more likely the SNP has damaging effect.;

1000g2015aug_all, 1000 Genomes Project; MutationTaster p-value (MTori), ranges from 0 to 1. Scores range from 0 to 1. The larger the score the more likely the SNP has damaging effect

ExAC_ALL, Exome Aggregation Consortium ExAC; CADD_phred, scores.The larger the score the more likely the SNP has damaging effect ;

ExAC_AFR, Exome Aggregation Consortium ExAC African/African American;

ExAC_AMR, Exome Aggregation Consortium ExAC Latino ExAC;

ExAC_EAS, Exome Aggregation Consortium ExAC East Asian ExAC;

ExAC_FIN, Exome Aggregation Consortium ExAC Finnish ExAC;

ExAC_NFE, Exome Aggregation Consortium ExAC Non-Finnish European ExAC;

ExAC_OTH, Exome Aggregation Consortium ExAC Other ExAC;

ExAC_SAS, Exome Aggregation Consortium ExAC South Asian ExAC;
