# Supplementary material for: Novel method for the genomic analysis of PKD1 mutation in autosomal dominant polycystic kidney disease
Source: Front Cell Dev Biol. 2023 Jan 9;10:937580. doi: 10.3389/fcell.2022.937580 (PMC9868468; doi:10.3389/fcell.2022.937580)
Supplement: Supplementary file 4 [file Table9.DOCX]

| Gene | Transcript | Nucleotide changes | Amino acids |
| --- | --- | --- | --- |
| *PKD1* | NM_001009944; | c.151T>C | p.C51R |
| *PKD1* | NM_001009944 | c.61dupG | p.A21Gfs93 |
| *PKD1* | NM_001009944 | c.2180T>C | p.L727P |

Supplementary table 9. all the mutations in the study
