# Supplementary material for: Novel method for the genomic analysis of PKD1 mutation in autosomal dominant polycystic kidney disease
Source: Front Cell Dev Biol. 2023 Jan 9;10:937580. doi: 10.3389/fcell.2022.937580 (PMC9868468; doi:10.3389/fcell.2022.937580)
Supplement: Supplementary file 5 [file Table2.DOCX]

Supplementary table 2 Primary PCR amplification and the PCR program：

| Step | Temperature | Time | Cycle |
| --- | --- | --- | --- |
| 1 | 98℃ | 3 min | 1 |
| 2 | 98℃ | 20 s | 3X |
|  | 70℃ | 30 s |  |
|  | 72℃ | 30 s |  |
| 3 | 98℃ | 20 s | 5X |
|  | 68℃ | 30 s |  |
|  | 72℃ | 30 s |  |
| 4 | 98℃ | 20 s | 8X |
|  | 65℃ | 30 s |  |
|  | 72℃ | 30 s |  |
| 5 | 98℃ | 20 s | 10X |
|  | 63℃ | 30 s |  |
|  | 72℃ | 30 s |  |
| 6 | 98℃ | 20 s | 15X |
|  | 61℃ | 30 s |  |
|  | 72℃ | 30 s |  |
| 7 | 72℃ | 5 min | 1 |
| 8 | 4℃ | Hold | 1 |
