# Supplementary material for: Novel method for the genomic analysis of PKD1 mutation in autosomal dominant polycystic kidney disease
Source: Front Cell Dev Biol. 2023 Jan 9;10:937580. doi: 10.3389/fcell.2022.937580 (PMC9868468; doi:10.3389/fcell.2022.937580)
Supplement: Supplementary file 7 [file Table3.DOCX]

Supplementary table 3 Secondary PCR amplification and amplification system

| Reagent | Volume（μL） |
| --- | --- |
| 5X Phusion HF Buffer | 5 |
| dNTP (10mM） | 0.6 |
| PE1.0 (50μM) | 1 |
| Barcode (50μM) | 1 |
| The product was purified by PCR in previous step | 2 |
| Phusion Hot Start II DNA Polymerase | 0.5 |
| ddH2O | Up to 25 μL |
