# Supplementary figures and images for: Novel method for the genomic analysis of PKD1 mutation in autosomal dominant polycystic kidney disease
Source: Front Cell Dev Biol. 2023 Jan 9;10:937580. doi: 10.3389/fcell.2022.937580 (PMC9868468; doi:10.3389/fcell.2022.937580)

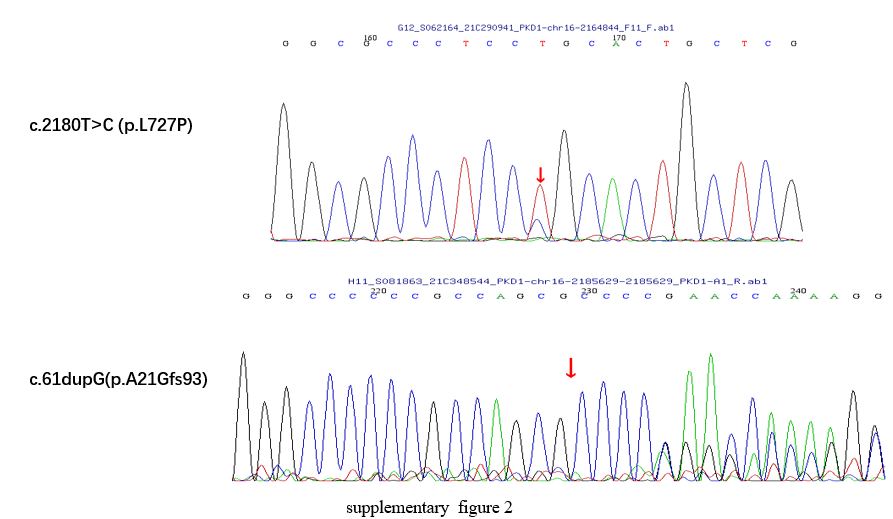

Supplement: Supplementary file 8 [file Image2.PNG]

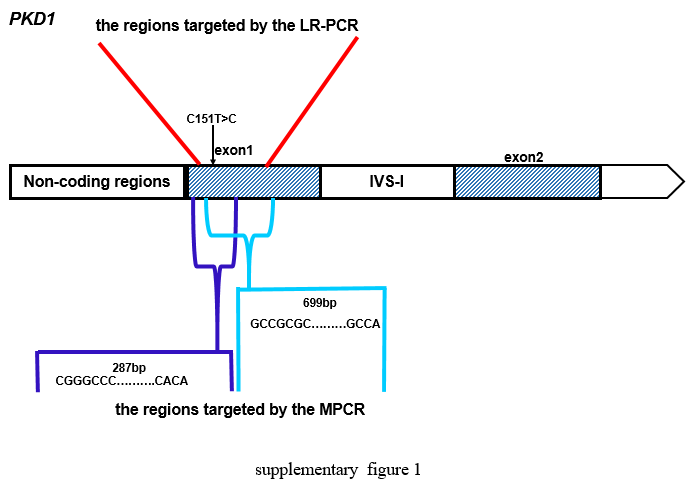

Supplement: Supplementary file 10 [file Image1.PNG]
