# Supplementary material for: Novel method for the genomic analysis of PKD1 mutation in autosomal dominant polycystic kidney disease
Source: Front Cell Dev Biol. 2023 Jan 9;10:937580. doi: 10.3389/fcell.2022.937580 (PMC9868468; doi:10.3389/fcell.2022.937580)
Supplement: Supplementary file 9 [file Table4.DOCX]

Supplementary table 4 Secondary PCR amplification and The PCR program

| Step | Temperature | Time | Cycle |
| --- | --- | --- | --- |
| 1 | 98℃ | 2 min |  |
| 2 | 98℃ | 20 sec | 6 cycles |
|  | 65℃ | 30 sec |  |
|  | 72℃ | 30 sec |  |
| 3 | 72℃ | 5 min |  |
| 4 | 4℃ | Hold |  |
