# Supplementary material for: Novel method for the genomic analysis of PKD1 mutation in autosomal dominant polycystic kidney disease
Source: Front Cell Dev Biol. 2023 Jan 9;10:937580. doi: 10.3389/fcell.2022.937580 (PMC9868468; doi:10.3389/fcell.2022.937580)
Supplement: Supplementary file 11 [file Table6.DOCX]

The second amplicon of the MPCR/targeted region (699bp):

GCCGCGCCGGAGCCTCGCCCTGGGAGCGTCCTGGCCCGCGTCCTGCTTCCCGTCCCGGGCCAGGGAACGCGCCCACGCCCGCCCGTCCCGCGGCCTCTCCCGGGTGCCGCTGGGCCCGCTACTCACAGCGCTGTGGCGTCCGCGGGGATGCGCAGCGCGGGACCGAGCGTCCGCAGCCCGCGGCCCGAGCAGTTGACGCGGCAGGCGGCGCCGGGCGCTGGGCCGCAGAGGCAGGGGGGCTCGCAGGGCCCGCAGCCGCGCCCGGGGCCCCCCGCCAGCGCCCCGAGCCACAGGCCCAGGCCCAGGGCCAGCGCCAGGCGGGCGGGCGCGGCGGGCGGCATCGTTAGGGCAGCGCGCGCATGGCCCCGCCGTCCCCAGGCCCGCCCGCGCGCGGAGGCCGCAGCTCAGGCGGGGCCCGCGGACGGCATGGCGGGCGCGGGGCTGGATGGGGCTGCGGCCGCGACCTGCTGCTGAGCGACGCCCGCTCGGGGCTCGGGGCCAGGCCGCTCCGGGAGCTCGGCCGCCCGCTCGGACGCTGGCGCTGCAGTGCGGGCCCCGCCGCGGCTCCTCCTCCTCCTCCCCGCGCGGCGCGGGGCGGACGGGGCGAGGGGGGGCGGGGCGGGTGCAGGCTCCGCCCCCTTCGCCACAGCGCGACCGGGCCA
